# From rumor to genetic mutation detection with explanations: a GAN approach

**DOI:** 10.1038/s41598-021-84993-1

**Published:** 2021-03-12

**Authors:** Mingxi Cheng, Yizhi Li, Shahin Nazarian, Paul Bogdan

**Affiliations:** 1 Ming Hsieh Department of Electrical and Computer Engineering, University of Southern California, Los Angeles, CA 90007 USA; 2 School of Computer Science, Beijing University of Posts and Telecommunications, Beijing, 100876 China

**Keywords:** Computer science, Mathematics and computing

## Abstract

Social media have emerged as increasingly popular means and environments for information gathering and propagation. This vigorous growth of social media contributed not only to a pandemic (fast-spreading and far-reaching) of rumors and misinformation, but also to an urgent need for text-based rumor detection strategies. To speed up the detection of misinformation, traditional rumor detection methods based on hand-crafted feature selection need to be replaced by automatic artificial intelligence (AI) approaches. AI decision making systems require to provide explanations in order to assure users of their trustworthiness. Inspired by the thriving development of generative adversarial networks (GANs) on text applications, we propose a GAN-based layered model for rumor detection with explanations. To demonstrate the universality of the proposed approach, we demonstrate its benefits on a gene classification with mutation detection case study. Similarly to the rumor detection, the gene classification can also be formulated as a text-based classification problem. Unlike fake news detection that needs a previously collected verified news database, our model provides explanations in rumor detection based on tweet-level texts only without referring to a verified news database. The layered structure of both generative and discriminative models contributes to the outstanding performance. The layered generators produce rumors by intelligently inserting controversial information in non-rumors, and force the layered discriminators to detect detailed glitches and deduce exactly which parts in the sentence are problematic. On average, in the rumor detection task, our proposed model outperforms state-of-the-art baselines on PHEME dataset by $26.85\%$ in terms of macro-f1. The excellent performance of our model for textural sequences is also demonstrated by the gene mutation case study on which it achieves $72.69\%$ macro-f1 score.

## Introduction

Sequential synthetic data generation such as generating text and images that are indistinguishable to human eyes have become an important problem in the era of artificial intelligence (AI). Generative models, e.g., variational autoencoders (VAEs)^1^, generative adversarial networks (GANs)^2^, recurrent neural networks (RNNs) with long short-term memory (LSTM) cells^3^, have shown outstanding generation power of fake faces, fake videos, etc. GANs as one of the most powerful generative models estimate generative models via an adversarial training process^2^. Real-valued generative models have found applications in image and video generation. However, GANs face challenges when the goal is to generate sequences of discrete tokens such as text^4^. Given the discrete nature of text, backpropagating the gradient from the discriminator to the generator becomes infeasible^5^. Training instability is a common problem of GANs, especially those with discrete settings. Unlike image generation, the autoregressive property in text generation exacerbates the training instability since the loss from discriminator is only observed after a sentence has been generated completely^5^. To remedy some of these difficulties, several AI approaches (e.g., Gumbel-softmax^6,7^, Wasserstein GAN (WGAN)^8,9^, reinforcement learning (RL)^4,10^) have been proposed^11,12^. For instance, the Gumble-softmax uses a reparameterization trick and softmax calculation to approximate the undifferentiable sampling operation on the generator output, which allows the model to perform backward propagation as well as provide discrete outputs approximating to actual values. GANs with Gumbel-softmax take the first step to generate very short sequences of small vocabulary^7^. WGAN method for discrete data directly calculates Wasserstein divergence between discrete labels and generator’s output as the criterion of discriminator. As a result, WGAN models can update parameters to learn the distribution of discrete data and produce some short sentences in character-level^9^. As a result, generating natural language-level sentences is still non-trivial. GANs with RL can skirt the problem of information loss in the data conversion by modeling text generation as a sequence of decisions and update the generator with reward function. Comparing to previous methods, RL can help GANs generate interpretable text closer to natural language^4^. In addition to the recent development in GAN-based text generation, discriminator-oriented GAN-style approaches are proposed for detection and classification applications, such as rumor detection^13^. Differently from the original generator-oriented GANs, discriminator-oriented GAN-based models take real data (instead of noise) as the input to the generator. Fundamentally, the detector may get high performance through the adversarial training technique. Current adversarial training strategies improve the robustness against adversarial samples. However, these methods lead to reduction of accuracy when the input samples are clean^14^.

Social media and micro-blogging have become increasingly popular^15,16^. The convenient and fast-spreading nature of micro-blogs fosters the emergence of various rumors. Social media rumors / misinformation / fake news are major concerns especially during major events, such as the global rise of COVID-19 and the U.S. presidential election. Some of the coronavirus rumors have been verified later to be very dangerous false claims, e.g., “those that suggest drinking bleach cures the illness”^17^ have made social media companies such as Facebook to find more effective solutions^18^. Commercial giants, government authorities, and academic researchers take great effort in diminishing the negative impacts of rumors^19^. Rumor detection has been formulated into a binary classification problem by a lot of researchers. Traditional approaches based on hand-crafted features describe the distribution of rumors^20,21^. However, early works depending on hand-crafted features require heavy engineering skills. More recently, with the rise of deep learning architectures, deep neural network (DNN)-based methods extract and learn features automatically, and achieve significantly high accuracies on rumor detection^22^. Generative models have also been used to improve the performance of rumor detectors^13^, and formulate multi-task rumor classification systems^23^ to realize rumor detection, tracking, stance and veracity classification. However, binary rumor classification lacks explanation since it only provides a binary result without expressing which parts of a sentence could be the source of the problem. The majority of the literature defines rumors as “an item of circulating information whose veracity status is yet to be verified at the time of posting”^24^. Providing explanations is challenging for detectors working on unverified rumors. Comparably, fake news is more well-studied, as it has a verified veracity. Attribute information, linguistic features, and semantic meaning of post^25^ and/or comments^26^ have been used to provide explainability for fake news detection. A verified news database has to be established for these approaches. However, for rumor detection, sometimes a decision has to be made based on the current tweet only. Text-level models with explanations that recognize rumors by feature extraction should be developed to tackle this problem.

Gene classification and mutation detection usually work with textual-gene data and also relate to a broad range of real-world applications, such as gene-disease association, genetic disorder prediction, gene expression classification, and gene selection. Machine learning-based classification and prediction tools have been proposed to solve these genetic problems^27,28^. Since essentially a gene sequence is of textual nature, we can process a genetic sequence as text. Gene mutation detection looks for abnormal places in a gene sequence^29^. Hence, we propose to solve this problem by using a natural language processing-based mutation detection model. When comparing a gene sequence with a natural language sequence, we observe that the mutations in genetic sequences represent abnormalities that makes the sequence do not fit well compared to other sequences from a biological perspective. The known genetic mutation detection and classification problem has been effectively explored in the literature, while the unknown mutation detection and classification has remained as a harder problem in both medical and machine learning fields. To detect unknown mutations and classify them, we propose a GAN-based framework that maintains a high performance level while facing unseen data with unknown patterns and providing explainability capabilities.

In this work, we propose a GAN-based layered framework that overcomes the afore-mentioned technical difficulties and provides solutions to (1) text-level rumor detection with explanations and (2) gene classification with mutation detection. In terms of solving the technical difficulties, our model keeps the ability of discriminating between real-world and generated samples, and also serves as a discriminator-oriented model that classifies real-world and generated fake samples. We overcome the infeasibility of propagating the gradient from discriminator back to the generator by applying policy gradient similar to SeqGAN^4^ to train the layered generators. In contrast to prior works, we adopt a RL approach in our framework because by combining the GAN and RL algorithmic strategies the framework can produce textural representations with higher quality and balance the adversarial training. The training instability of long sentence generation is lowered by selectively replacing words in the sentence. We solve the per time step error attribution difficulty by word-level generation and evaluation. We show that our model outperforms the baselines in terms of addressing the degraded accuracy problem with clean samples only.

Our GAN-based framework consists of a layered generative model and a layered discriminative model. The generative model generates high-quality sequences by first intelligently selecting items to be replaced, then choosing appropriate substitutes to replace those items. The discriminative model provides classification output with explanations. For example, in the gene classification and mutation detection task, the generative model mutates part of the genetic sequence and then the discriminative model classifies this genetic sequence and tells which genes are mutated. The major contributions of this work are: (1) this work delivers an explainable rumor detection without requiring a verified news database. Rumors could stay unverified for a long period of time because of information insufficiency. Providing explanations of which words in the sentence are problematic is critical especially when there is no verified fact. When a verified news database is achievable, our model is capable to realize fake news detection with minor modifications. (2) Our model is a powerful textural mutation detection framework. We demonstrate the mutation detection power by applying our proposed model to the task of gene classification with mutation detection. Our model accurately identifies tokens in the gene sequences that are exibiting mutations, and classifies mutated gene sequences with high precision. (3) The layered structure of our proposed model avoids the function mixture and boosts the performance. We have verified that using one layer to realize two functions either in generative or discriminative model causes function mixture and hurts the performance.

## Results

### Rumor detection with explanations

Rumors, defined as “items of circulating information whose veracity status is yet to be verified at the time of posting”^24^, usually emerge when there are influential events and spread rapidly with the rise of social media. Far-reaching and fast-spreading rumors can cause serious consequences, for example, they are growing threats to the democratic process^30^. Rumor detection suffers from the limitation of datasets scale and the uncertain nature of rumors makes the early-detection and classification with explanation challenging. In this section, the proposed discriminator-oriented GAN framework utilizes the layered generative model to generate augmented rumor dataset, and uses $D_{classify}$ to classify a rumor while relying on $D_{explain}$ to indicate which parts of the sentence are suspicious. The detailed model description can be found in “Methods” section.

#### Detection results

**Figure 1:**
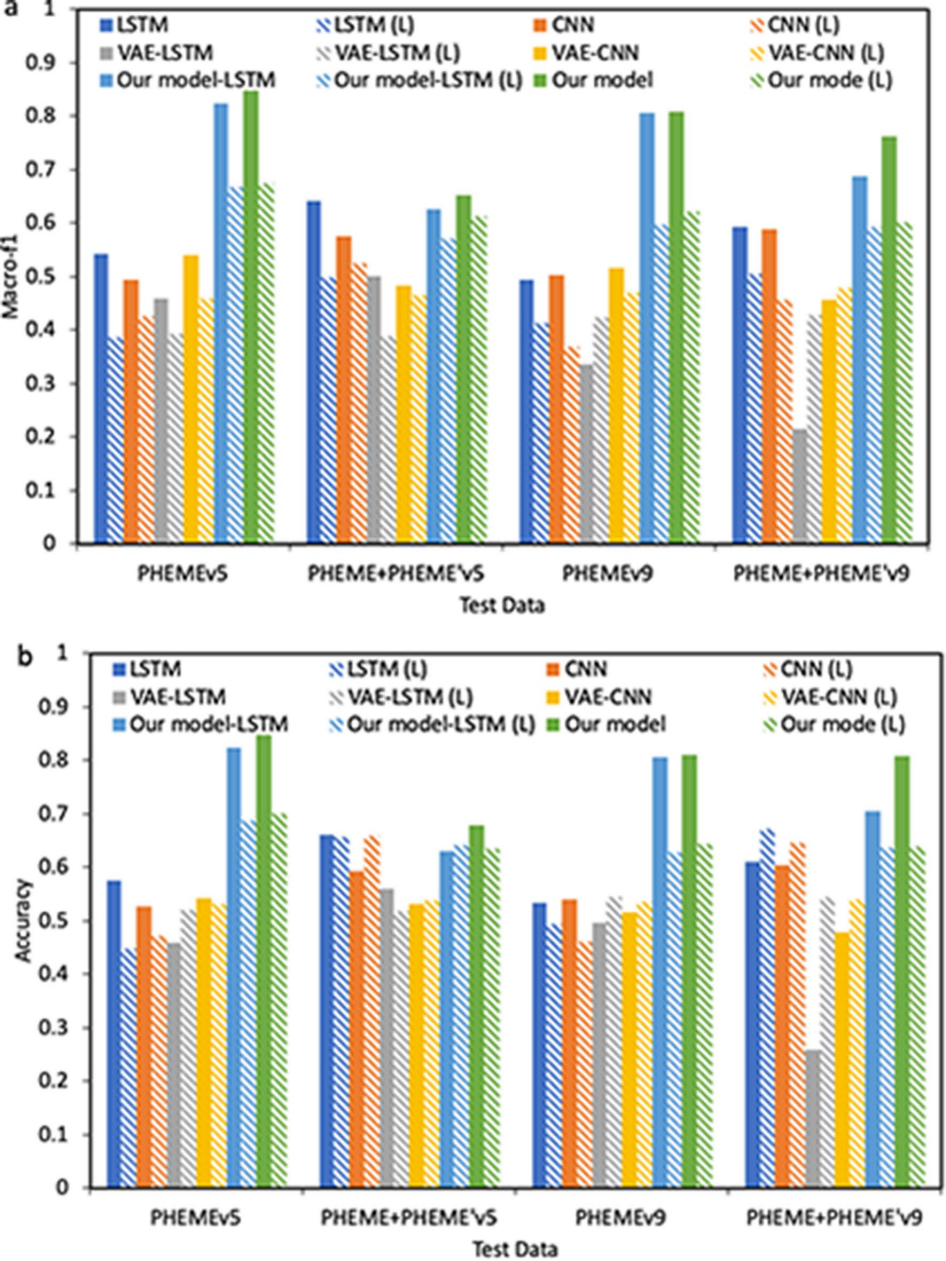
Macro-f1 (**a**) and accuracy (**b**) comparison between our model (-CNN and our model-LSTM) and baselines on the rumor detection task. The models are trained on augmented dataset PHEME+PHEME’ and tested on both original PHEME and augmented PHEME+PHEME’. L represents the model is evaluated under leave-one-out principle.

Table 1 and Fig. 1 illustrate a comparison between the proposed model $D_{classify}$ and the baselines for rumor detection. In this experiment, we use PHEME data to train our model. During training, our model generates PHEME’ to enhance the discriminative model. Data in PHEME are either rumor (*R*), or non-rumor (*N*), and generated data in PHEME’ are all labeled as *R* since we would like our $D_{classify}$ to be conservative and filter out human-written non-rumors. Hence, all models in Table 1 perform 2-class classification (*R*/*N*). In real world applications, the original clean dataset is available all the time. However, the modified or adversarial data that contains different patterns are not always accessible. Models like LSTM and CNN do not have generalization ability and usually perform worse facing adversarial input. Generative models such as GANs are more robust. In VAE-LSTM and VAE-CNN, we first pre-train VAEs, then LSTM and CNN are trained under latent representations of pre-trained VAEs. Under the first evaluation principle, our model and the variation of our model with LSTM cells outperform all baselines in terms of both macro-f1 and accuracy. Accuracy is not sufficient when the test data are not balanced, hence macro-f1 is provided for comprehensive comparison. Under the first evaluation principle, the robustness and generalization ability of our model are shown by comparing with baselines under PHEME+PHEME’. Our model reaches the highest values in both versions of PHEME+PHEME’ and the variation of our model with LSTM cells follows as the second best. Under leave-one-out (L) principle (i.e., leave out one news topic for test and use the rest for training), our proposed model and the variation achieve the highest macro-f1 scores in all cases. These results confirm the rumor detection ability of the proposed layered structure under new, out-of-domain data. Adversarial training of baselines improves generalization and robustness under PHEME+PHEME’, but hurts the performance under clean data as expected. Although our model and the variation are trained adversarially, they achieve the highest macro-f1 under clean data PHEME. The results confirm that our model outperforms the baselines in terms of addressing the accuracy reduction problem.

**Table 1:** Macro-f1 and accuracy comparison between our model and baselines on the rumor detection task.

	PHEMEv5				PHEMEv9			
	PHEMEv5		PHEME+PHEME’v5		PHEMEv9		PHEME+PHEME’v9	
	Macro-f1	Accuracy	Macro-f1	Accuracy	Macro-f1	Accuracy	Macro-f1	Accuracy
LSTM	0.6425	0.6542	0.4344	0.4345	0.6261	0.6269	0.4999	0.5283
CNN	0.6608	0.6660	0.4792	0.4833	0.6549	0.6552	0.5028	0.5253
VAE-LSTM	0.4677	0.5625	0.2582	0.2871	0.4454	0.4589	0.4231	0.4326
VAE-CNN	0.5605	0.5605	0.4655	0.4902	0.3859	0.5029	0.2513	0.2778
GAN-GRU	$0.7810^*$	$0.7810^*$	–	–	–	–	–	–
Our model-LSTM	0.8242	0.8242	0.6259	0.6302	0.8066	0.8066	0.6884	0.7044
Our model-CNN	**0.8475**	**0.8476**	**0.6524**	**0.6777**	**0.8084**	**0.8095**	**0.7620**	**0.8085**
LSTM (L)	0.5693	0.6030	0.5260	0.5710	0.5217	0.5827	0.5055	0.5906
CNN (L)	0.5994	0.6406	0.5324	0.5779	0.5477	0.6035	0.5051	0.5769
VAE-LSTM (L)	0.3655	0.3996	0.3620	0.3959	0.4256	0.5367	0.4284	0.5397
VAE-CNN (L)	0.4807	0.5190	0.4816	0.5214	0.4316	0.4597	0.4314	0.4587
DATA-AUG (L)	$0.5350^*$	$\mathbf{0} .\mathbf{7070} ^*$	–	–	–	–	–	–
Our model-LSTM (L)	0.6666	0.6866	0.5703	$\mathbf{0} .\mathbf{6411}$	0.5972	0.6272	0.5922	0.6371
Our model-CNN (L)	$\mathbf{0} .\mathbf{6745}$	0.7016	$\mathbf{0} .\mathbf{6126}$	0.6342	$\mathbf{0} .\mathbf{6207}$	$\mathbf{0} .\mathbf{6438}$	$\mathbf{0} .\mathbf{6016}$	$\mathbf{0} .\mathbf{6400}$

The models are trained on PHEME and tested on both original dataset PHEME and augmented dataset PHEME+PHEME’. *indicates the best result from the work that proposed the corresponding model. L represents the model is evaluated under leave-one-out principle. Variance results in cross-validations are shown in Table 2.

The best results are marked in bold.

**Table 2:** Variance results in cross-validations on the rumor detection task.

Methods/variance	PHEMEv5		PHEME+PHEME’v5		PHEMEv9		PHEME+PHEME’v9	
	Macro-f1	Accuracy	Macro-f1	Accuracy	Macro-f1	Accuracy	Macro-f1	Accuracy
LSTM (L)	0.0028	0.0060	0.0003	0.0024	0.0262	0.0036	0.0022	0.0016
CNN (L)	0.0022	0.0013	0.0003	0.0012	0.0215	0.0048	0.0017	0.0015
VAE-LSTM (L)	0.0204	0.0086	0.0001	0.0006	0.0103	0.0082	0.0067	0.0013
VAE-CNN (L)	0.0037	0.0029	0.0013	0.0014	0.0006	0.0031	0.0020	0.0020
Our model-LSTM (L)	0.0022	0.0025	0.0015	0.0020	0.0095	0.0059	0.0093	0.0066
Our model-CNN (L)	0.0013	0.0023	0.0022	0.0029	0.0101	0.0048	0.0079	0.0051

**Table 3:** Examples of $D_{explain}$ and $D_{classify}$’s prediction on rumor (first) and non-rumor (second).

0.1579	**Who’s** your pick for worst **contribution** to sydneysiege **mamamia uber** or the daily tele
0.8558	Glad to hear the sydneysiege is over but saddened that it even happened to begin with my heart goes out to all those affected

The suspicious words in the rumor predicted by $D_{explain}$ are marked in bold. $D_{classify}$ provides a score ranging from 0 to 1. 0 and 1 represent rumor and non-rumor, respectively.

Table 3 shows two examples that are correctly detected by our model but incorrectly detected by other baselines. For the first rumor, baselines CNN, LSTM, VAE-CNN, and VAE-LSTM provide scores 0.9802, 0.9863, 0.4917, and 0.5138, respectively. Our model provides a very low score for a rumor, while other baselines all generated relatively high scores, and even detect it as non-rumor. This is a very difficult example since from the sentence itself, we as human rumor detection agents even cannot pick the suspicious parts confidently. However, our model gives a reasonable prediction and shows that it has the ability to understand and analyze complicated rumors. For the second non-rumor, baselines CNN, LSTM, VAE-CNN, and VAE-LSTM provide scores 0.0029, 0.1316, 0.6150, and 0.4768, respectively. In this case, a non-rumor sentence gains a high score from our model, but several relatively low scores from the baselines. This example again confirms that our proposed model indeed captures the complicated nature of rumors and non-rumors.

#### Explanation results

A component for decision explanation is realized by $D_{explain}$, which offers insight into the detection problem by suggesting suspicious parts of given rumor texts. Our model’s $D_{explain}$ recognizes the modified parts in sequences accurately. In 2-class PHEME experiments, its macro-f1 on PHEME’v5 and PHEME’v9 are $80.42\%$ and $81.23\%$, respectively. Examples of $D_{explain}$ predicting suspicious parts in rumors are shown in Table 4. In the first rumor, “hostage escape” is the most important part in the sentence, and if these two words are problematic, then the sentence is highly likely to be problematic. Given an unverified or even unverifiable rumor, $D_{explain}$ provides reasonable explanation without requiring a previously collected verified news database.

**Table 4:** Examples of $D_{explain}$ predicting suspicious words in rumors (marked in bold).

0.0010	Breaking update 2 **hostages escape** lindt café through front **door** 1 via fire door url sydneysiege url
0.0255	**Newest** putin **rumour** his girlfriend just gave birth to their child url cdnpoli russia
0.0300	Soldier **gets cpr** after being shot at war memorial in ottawa url
0.0465	Sydney’s **central business district** is under lockdown as gunman takes hostages at a cafe live **stream** as it unfolds url
0.2927	So in **5mins** mike brown **shaved** his head and changed his **scandals** to **shoes** i think your being lied too

$D_{classify}$ outputs probabilities in range [0, 1], where 0 and 1 represent rumor and non-rumor, respectively.

#### Rumor/non-rumor, true/false, and real/fake

Misinformation, disinformation, fake news, and rumor classifications have been studied in the literature^23,30–32^ and frequently suffer from small-scale datasets. The difference between misinformation, disinformation, fake news, and rumor is not well-defined and the labeling in these tasks is sometimes ambiguous and imprecise. In this work, we specifically refer rumor as a piece of information whose veracity is not verified, and its label in detection task is rumor (*R*)/non-rumor (*N*). With the consideration of veracity status, we refer facts as true (*T*) and false statements as false (*F*). Furthermore, we refer purely human-written statements as real (*E*) and machine-generated statements as fake (*K*). In the previous detection section, we do binary classification in rumor detection task. Our generative model replaces parts of a sequence and due to the uncertain nature of rumors, we label the generated (modified) rumors as *R*, and non-rumor in original dataset as *N* to emphasize the purpose of filtering out non-rumor in real-world applications. However, with real / fake and true/false labeling in misinformation or fake news classification, the labeling should be precise and 2-class labeling is not sufficient anymore for the generated (modified) sequences. Specifically, if an input sequence is labeled as *Y*, its modified version (i.e., the output of our generative model) is labeled as $Y'$ to represent that it is modified from a sequence with label *Y*. In what follows, we perform the following experiments: (1) rumor classification with PHEME again using 4-class labels: *R*, $R'$, *N*, $N'$; (2) misinformation (disinformation) classification with FMG (a misinformation/fake news dataset) using 4-class labels: *T*, $T'$, *F*, $F'$; and (3) fake news classification with FMG using 4-class labels: *E*, $E'$, *K*, $K'$.

Experimental results of PHEME (4-class) are shown in Table 5. Similar to previous PHEME experiment in Table 1, we generate a dataset PHEME’ to do data augmentation. However, different than before, this new generated PHEME’ (4-class) has four labels: *R*, $R'$, *N*, $N'$ and our GAN models are trained with 4-class classification. In addition, we train baselines with augmented dataset PHEME+PHEME’ (4-class) and test it with PHEME. Moreover, we find that training with augmented data improves the performance of baselines. Our models (-LSTM and -CNN) still provide best results compared to (augmented) baselines.

**Table 5:** Marco-f1 and accuracy comparison between our model and baselines on the extended 4-class experiments of rumor detection task on PHEME dataset.

	PHEMEv5				PHEMEv9			
	PHEMEv5 (2-class)		PHEME+PHEME’v5 (4-class)		PHEMEv9 (2-class)		PHEME+PHEME’v9 (4-class)	
	Macro-f1	Accuracy	Macro-f1	Accuracy	Macro-f1	Accuracy	Macro-f1	Accuracy
LSTM	0.6095	0.6259	0.2753	0.4121	0.6304	0.6484	0.2788	0.4179
LSTM (U)	0.6774	0.7480	0.5082	0.5073	0.6836	0.7446	0.5194	0.5205
CNN	0.6052	0.6210	0.2766	0.4135	0.6211	0.6396	0.2759	0.4135
CNN (U)	0.6760	0.7534	0.5109	0.5083	0.6678	0.7402	0.5239	0.5229
VAE-LSTM	0.5188	0.6591	0.2464	0.2753	0.4693	0.5205	0.1976	0.2416
VAE-LSTM (U)	0.4877	0.5810	0.2473	0.2578	0.4879	0.5351	0.2135	0.2602
VAE-CNN	0.4983	0.5629	0.2239	0.2529	0.4303	0.7495	0.1514	0.2504
VAE-CNN (U)	0.4912	0.5361	0.2566	0.2719	0.4813	0.5214	0.2160	0.2617
Our model-LSTM	$\mathbf{0} .\mathbf{7776}$	$\mathbf{0} .\mathbf{8271}$	$\mathbf{0} .\mathbf{5703}$	$\mathbf{0} .\mathbf{5678}$	$\mathbf{0} .\mathbf{7830}$	$\mathbf{0} .\mathbf{8339}$	$\mathbf{0} .\mathbf{5631}$	$\mathbf{0} .\mathbf{5610}$
Our model-CNN	0.7485	0.8017	0.5352	0.5419	0.7693	0.8232	0.5558	0.5600

U indicates that the model is trained on PHEME+PHEME’, otherwise it is train on original PHEME dataset. All models are tested on PHEME (*R*/*N*) and PHEME+PHEME’ (*R*/*N*/$R'$/$N'$).

The best results are marked in bold.

Besides rumor detection, we apply our framework in misinformation and fake news detection tasks using a fake news dataset (FMG)^33^, which includes both real/fake and true/false data. In real/fake task, models differentiate between purely human-written statements and (partially or fully) machine-generated statements, while in true/false task, models are required to identify true statements and false claims. We augment the original dataset (denoted as FMG) with our GAN-generated data (denoted as FMG’) and train several models with the augmented dataset (denoted as FMG+FMG’). Similarly in PHEME (4-class) experiments, we find that models trained with augmented FMG+FMG’ achieve higher performance on original FMG as shown in Table 6. From these experimental results, we conclude that our framework is effective in data augmentation and helps models to achieve higher accuracy. One thing to note is that in this experiment, our models do not outperform augmented LSTM and CNN in provenance classification task (although it is better than unaugmented ones). This could be due to the fact that the nature of provenance classification is to distinguish patterns between human-written and machine-generated sentences. In the early training process of our model, the training data (generated sequences) of our discriminative model are low-quality since the generative model is not well-trained. The generated sequences contain our machine-generated noisy patterns, which could make our model converge to suboptimal results.

**Table 6:** Marco-f1 and accuracy comparison between our model and baselines on the extended 4-class experiments of provenance (real/fake) and veracity (true/false) tasks.

	Provenance				Veracity			
	FMG (*E* / *K*)		FMG+FMG’ (4-class)		FMG (*T* / *F*)		FMG+FMG’ (4-class)	
	Macro-f1	Accuracy	Macro-f1	Accuracy	Macro-f1	Accuracy	Macro-f1	Accuracy
LSTM	0.3963	0.3965	0.2752	0.3745	0.4786	0.4890	0.1792	0.2739
LSTM (U)	0.7062	$\mathbf{0} .\mathbf{7989}$	$\mathbf{0} .\mathbf{6401}$	$\mathbf{0} .\mathbf{6450}$	0.6339	0.7689	0.4985	0.5194
CNN	0.3964	0.3965	0.2738	0.3730	0.5478	0.6352	0.1940	0.2984
CNN (U)	$\mathbf{0} .\mathbf{7082}$	0.7824	0.6287	0.6325	0.6802	0.7724	0.5392	0.5613
VAE-LSTM	0.4967	0.6305	0.2137	0.2288	0.5099	0.6175	0.2268	0.2740
VAE-LSTM (U)	0.4871	0.6910	0.2630	0.2797	0.5105	0.6172	0.2793	0.2920
VAE-CNN	0.4624	0.5055	0.2207	0.2494	0.4676	0.4989	0.2075	0.2495
VAE-CNN (U)	0.5122	0.6158	0.2607	0.2615	0.5013	0.6007	0.2644	0.2650
Our model-LSTM	0.6562	0.7529	0.5027	0.5054	0.6560	0.7524	0.5027	0.5054
Our model-CNN	0.5639	0.6984	0.4543	0.4615	$\mathbf{0} .\mathbf{7134}$	$\mathbf{0} .\mathbf{7779}$	$\mathbf{0} .\mathbf{5637}$	$\mathbf{0} .\mathbf{5673}$

U indicates that the model is trained on FMG+FMG’, otherwise it is train on FMG. All models are tested on FMG and FMG+FMG’.

The best results are marked in bold.

**Table 7:** Examples of $D_{explain}$ failing to predict suspicious words in some short rumors.

0.0112	Ottawa police report a third shooting at rideau centre no reports of injuries
0.0118	Breaking swiss art museum accepts artworks bequeathed by late art dealer gurlitt url
0.0361	Breaking germanwings co pilot was muslim convert url
0.4451	Germanwings passenger plane crashes in france url
0.5771	The woman injured last night ferguson url

$D_{classify}$ outputs probabilities in range [0, 1], where 0 and 1 represent rumor and non-rumor, respectively.

#### Limitations and error cases in rumor detection

Examples of error cases of our model in rumor detection task are presented in Table 7. For some short sentences, $D_{explain}$ sometimes fails to predict the suspicious parts. The reason is that the majority of training data are long sentences, hence $D_{explain}$ performs better with long sentences. We can solve this problem by feeding more short sentences to our model. In most cases, although $D_{explain}$ does not generate predictions, $D_{classify}$ still can provide accurate classification. As shown in Table 7, $D_{classify}$ outputs low score, i.e., classifies the input as rumor, for four out of five rumors.

### Gene classification with mutation detection

Genetic sequence classifications, gene mutation detection/prediction, DNA / RNA classification all work with genetic sequences, and deep learning-based methods in the literature take sequential data as input, and output the classification results^27,28,34^. Since our proposed framework demonstrates very good results for sequential / textural data (as shown in previous sections), next, we adopt a textural representation^35,36^ of gene sequences and investigate a gene mutation phenomenon. Note that binary format representation of genetic sequences is also frequently used in the literature^37,38^. In our GAN framework, the input to the models is first encoded into a high-dimensional vector, therefore, the binary formatting does not affect the experimental results. In this experiment, we first perform a mutation in genetic sequences by the generative model, and then use $D_{classify}$ to classify a genetic sequence and predict which parts of the sequence is mutated. We find that our framework not only provides high accuracy in classification task, but also accurately identifies the mutations in the generated sequences.

In this experiment, all models are trained under NN269+NN269’ (an augmented dataset) to ensure fairness, and we follow the labeling rule in misinformation/fake news detection task. When testing with NN269+NN269’, there are 8 classes in total: *AP*, *AN*, *DP*, *DN* from NN269 (original splice site dataset) and $AP'$, $AN'$, $DP'$, $DN'$ from NN269’ (generated dataset). Detailed experiment setup can be found in “Methods” section. If solely clean data from NN269 is accessible during training, then our proposed model and the variation of our proposed model are the only models that can recognize if a given sequence is modified or unmodified. Comparison between our model’s (and the variation’s) $D_{classify}$ and baselines is shown in Table 8. Under long acceptor data, baselines perform significantly worse than our model and the variation. Under short donor data, our model and the variation achieve highest AURoCs. This implies that our model and the variation are stronger when the input are long sequences. The layered structure and adversarial training under the augmented dataset provide our model the ability of extracting meaningful patterns from long sequences. For short sequences, our model and the variation provide highest AURoC, and simpler models such as CNN can also give good classification results. This is because for short sequences, textural feature mining and understanding is relatively easier then in long sequences. Under NN269’, our model’s $D_{classify}$ and $D_{explain}$ achieve $92.25\%$ and $72.69\%$ macro-f1, respectively. Examples of $D_{explain}$’s prediction are shown in Table 9. The results suggest that our model can not only classify a gene-sequence, but also provide an accurate prediction that explains which part of the sequence is modified.

**Table 8:** Comparison between our model and baselines on the gene classification with the mutation detection task.

	NN269 (2-class)			NN269+NN269’ (2-class)			NN269+NN269’ (4-class)		
	Macro-f1	Accuracy	AURoC	Macro-f1	Accuracy	AURoC	Macro-f1	Accuracy	AURoC
LSTM (A)	0.8120	0.8870	0.9305	0.7794	0.8580	0.9036	0.7800	0.8580	0.9715
CNN (A)	0.5663	0.7933	0.6324	0.5594	0.7808	0.6131	0.5593	0.7808	0.8875
VAE-LSTM (A)	0.7664	0.8566	0.8451	0.6781	0.8323	0.7780	0.6531	0.8342	0.8806
VAE-CNN (A)	0.5657	0.7539	0.6135	0.5744	0.7651	0.6219	0.5379	0.7470	0.8411
EFFECT (A)	–	–	$0.9770^*$	–	–	–	–	–	–
Our model-LSTM (A)	0.9131	0.9458	0.9781	0.8794	0.9243	0.9658	0.8758	0.9223	0.9879
Our model-CNN (A)	$\mathbf{0} .\mathbf{9175}$	$\mathbf{0} .\mathbf{9494}$	$\mathbf{0} .\mathbf{9807}$	$\mathbf{0} .\mathbf{8831}$	$\mathbf{0} .\mathbf{9301}$	$\mathbf{0} .\mathbf{9691}$	$\mathbf{0} .\mathbf{8839}$	$\mathbf{0} .\mathbf{9311}$	$\mathbf{0} .\mathbf{9894}$
LSTM (D)	0.8336	0.8214	0.9003	0.8148	0.7998	0.8802	0.7648	0.7530	0.9246
CNN (D)	0.9131	0.9393	0.9795	$\mathbf{0} .\mathbf{9025}$	$\mathbf{0} .\mathbf{9323}$	0.9746	$\mathbf{0} .\mathbf{8336}$	$\mathbf{0} .\mathbf{8583}$	0.9596
VAE-LSTM (D)	0.8011	0.8515	0.9218	0.7336	0.8329	0.8217	0.5774	0.7692	0.9194
VAE-CNN (D)	0.8386	0.8772	0.9554	0.7909	0.8593	0.8528	0.5585	0.7415	0.9190
EFFECT (D)	–	–	$0.9820^*$	–	–	–	–	–	–
Our model-LSTM (D)	0.9272	0.9484	$\mathbf{0} .\mathbf{9822}$	0.8802	0.9140	$\mathbf{0} .\mathbf{9766}$	0.8113	0.8580	0.9541
Our model-CNN (D)	$\mathbf{0} .\mathbf{9274}$	$\mathbf{0} .\mathbf{9494}$	0.9810	0.8988	0.9296	0.9635	0.8119	0.8470	$\mathbf{0} .\mathbf{9776}$

*The best result from the corresponding paper. 2-class refers to *AP*, *AN* for acceptor, and *DP*, *DN* for donor. 4-class refers to *AP*, *AN*, $AP'$, $AN'$ for acceptor, and *DP*, *DN*, $DP'$, $DN'$ for donor. A and D indicate acceptor and donor.

The best results are marked in bold.

**Table 9:** Examples of the generative model modifying gene sequences and the discriminative model detecting the modifications (marked in bold).

Original	*GGTGGGTGTAGCCGTGGCTAGGGCTGACGGGGCCACTTGGGCTTGGCCGCATGCCCCTGTGCCCCACCAGCCATCCTGAACCCAACCTAG*
Modified	$G G T G G G T G T A G C C G T G G C T A G G G C T G A C G G G G C C A C T T G G G C T T G G C \mathbf{A} G C A T G \mathbf{NNN} C T G T G C C C C A C C A G C C A T \mathbf{G} C T G A A C C C A A C C T A G$
Prediction	$\mathbf{G} G T G G G T G T A G C C G T G G C T A G G G C T G A C G G G G C C A C T T G G G C T T G G C A G C A T G \mathbf{NNN} C T G T G C C C C A C C A G C C A T G C T G A A C C C A A C C T A G$
Original	*GCGCGGGGCGCTGAGCTCCAGGTAGGGCGCGCAGCCTGGTCAGGTGGCAGCCTTACCTCAGGAGGCTCAGCAGGGGTCCTCCCCACCTGC*
Modified	$G C G C G G G G C G C T G A G C T C C A G G T A G G G C G C G C A G C C T G G T C A G G T G G C A G \mathbf{GN} T T A \mathbf{TS} T C A G G A G G C T C A G C A G G G G T C \mathbf{A} T C C C C A C C T G C$
Prediction	$G C G C G G G G C G C T G A G C T C C A G G T A G G G C G C G C A G C C T G G T C A G G T G G C A G G \mathbf{N} T \mathbf{T} A T \mathbf{ST} C A G G A G G C T C A G C A G G G G T C A T C C C C A C C T G C$
Original	*TGGTGGCTAATTCAGGAATGTGCTGCTGTCTTTCTGCAGACGGGGGCAAGCACGTGGCATACATCATCAGGTCGCACGTGAAGGACCACT*
Modified	$T G G T G G C T A A T T C A G G A A T G T G \mathbf{N} T G \mathbf{N} T G T \mathbf{S} T T T \mathbf{G} T G C A G A C G G G G G C A A G C A C G T G G C A T A C A T C A T C A G G T \mathbf{N} G C A C G T G A A G G A C C A C T$
Prediction	$T G G T G G C T A A T T C A G G A A T G T G \mathbf{N} T G \mathbf{N} T G T S T T T G \mathbf{T} G C A G A C G G G G G C A A G C A C G T G G C A T A C A T C A T C A G G T \mathbf{N} G C A C G T G A A G G A C C A C T$

## Discussion

Rumor, as a piece of circulating information without verified veracity status, is hard to detect, especially when we have to point out why it is a rumor. Misinformation, whose veracity is determined, can be detected where there exists a verified database containing information about why the misinformation is wrong. Rumor detection is a hard problem and rumor detectors in the literature usually suffer from the low accuracy. The reason for unsatisfactory performance is multi-fold: for example, rumor dataset is usually small and imbalanced. The data-driven machine learning detectors don’t have sufficient high-quality data to work with, hence the data shortage causes the low or extremely imbalanced performance. Rumors usually emerge violently during emergent national or even international events and confirming the veracity of rumors can take a long time and an aggressive amount of human resource. Therefore, rumors could stay as floating and circulating pieces of information without veracity confirmed for a long time and provoke social panic, such as in the recent coronavirus breakout events. Rumors are associated with different events, so if the detector is trained with previously observed rumors on other events, the detection of current unseen rumors associated with the new event usually results in low accuracy because the patterns of the rumors are changed. Compared to the detection problem, pointing out the problematic parts of the rumors is even more difficult due to the similar reasons.

We propose a framework that addresses the afore-mentioned issues. To solve the limited and imbalance data issue and the low performance problem, our proposed GAN-based framework augments the dataset by generating new rumors/misinformation/fake news and uses the augmented data to train the discriminators to achieve high accuracy. The layered generative model intelligently decides about where and how to modify the input sequences. This process injects noise in data and pushes the discriminators to learn the essential semantic and syntactic features of the rumors. Therefore, this process alleviates the impact of event-associated patterns. To provide reasonable explanations of why the sentence is potentially a rumor, we improve the discriminator in GAN to include a layered structure to (1) make the detection decision, (2) generate the explanation, and (3) provide a corresponding layered model-tuning signal to the layered generative model.

Genetic sequences classification, genetic mutation detection/prediction, gene-disease association, and DNA expression classification all work with gene sequences. Machine learning-based methods such as support vector machines and deep neural networks have already been used to solve these problems. We propose and verify the applicability of our designed framework on gene classification and mutation detection in this work. The fundamental rationality comes from that the genetic sequence essentially is textual data. Since our proposed framework is aiming to take textual data as input and make classification decisions, it is reasonable to apply the framework to gene data. Mutation detection in gene data is to find the abnormal places in a gene sequence and rumor detection with explanation is to find the abnormal places in a sentence. One problem facing by gene mutation detection is that there might be some unknown patterns in the gene sequence, which is similar to the generalization problem in rumor detection: unknown patterns exist in unobserved rumors. Hence, our proposed GAN-based model can alleviate this issue by intelligently augmenting the dataset. From an algorithmic perspective, the problem of rumor detection and gene classification can be formulated as a textual sequence classification problem. (Although genetic sequence representation can be in binary format, we have discussed that binary formatted genetic sequences can be further encoded into vectors as the input to our model, which does not generate different results in our experiments). Therefore, our framework as a sequential data classification model should be applicable to both rumor and gene classification. We can learn which parts are suspicious/machine generated in a rumor, and this is no different than given a sequence, we learn which parts contain abnormal patterns. Following similar reasoning, in gene mutation detection task, our model learns which parts in a genetic sequence are abnormal. The difference is that language has intuitive semantic meanings, however, genetic sequence may have unknown hidden semantic meanings. Our goal is to investigate them both even though are different in order to provide this as an example of a methodology for interdisciplinary research and analysis.

In summary, we proposed a layered text-level rumor detector and gene mutation detector with explanations based on GAN. We used the policy gradient method to effectively train the layered generators. Our proposed model outperforms the baseline models in mitigating the accuracy reduction problem, that exists in case of only clean data. We demonstrate the classification ability and generalization power of our model by comparing with multiple state-of-the-art models in both rumor detection and gene classification with mutation detection problems. On average, in the 2-class rumor detection task, our proposed model outperforms the baselines on clean dataset PHEME and enhanced dataset PHEME+PHEME’ by $26.85\%$ and $17.04\%$ in terms of macro-f1, respectively. Our model provides reasonable explanation without a previously constructed verified news database, and achieves significantly high performance. In the gene classification with mutation detection task, our model identifies the mutated gene sequence with high precision. On average, our model outperforms baselines in both NN269 and NN269+NN269’ (2-class) by $10.71\%$ and $16.06\%$ in terms of AURoC, respectively. In both rumor detection and gene mutation detection tasks, our model’s ability of explanation generation is demonstrated by identifying the mutations accurately (above $70\%$ macro-f1). We find that using two discriminators to perform classification and explanation separately achieves higher performance than using one discriminator to realize both functions. We also found the pre-train of $D_{classify}$ and varying $N_{replace}$ contribute to the high accuracy of $D_{explain}$.

Despite the high performance in both applications, we do find a limitation of our framework. $D_{explain}$ sometimes fails to provide explanations in rumor experiments when the input sentences are very short, even though the corresponding $D_{classify}$ generates accurate predictions. One potential reason for this result is that the dataset contains a small number of short sentences and the model is not trained enough in short sentence cases. We also observed $D_{explain}$ performs a bit worse in gene mutation detection experiments than in rumor detection task. It could be caused by the choice of $N_{replace}$ (the number of items to be replaced in a sequence), which is a hyper parameter that affects the mutation detection ability. As part of our future work, to improve the performance of the discriminators, we would like to choose $N_{replace}$ intelligently. To enhance the performance of our generators, we would like to explore the application of hierarchical attention network^39^. We will also investigate the dependencies between the discriminators of our model to benefit $D_{explain}$ from the accurate $D_{classify}$.

We believe our proposed framework could be beneficial to numerous textual data-based problems, such as rumor and misinformation detection, review classification for product recommendation, twitter-bot detection and tracking, false information generation and attack defense, and various genetic data-based applications. We connect the genetic data processing and the natural language processing field and provide new angles and opportunities for researchers in both fields to contribute mutually.

## Methods

**Figure 2:**
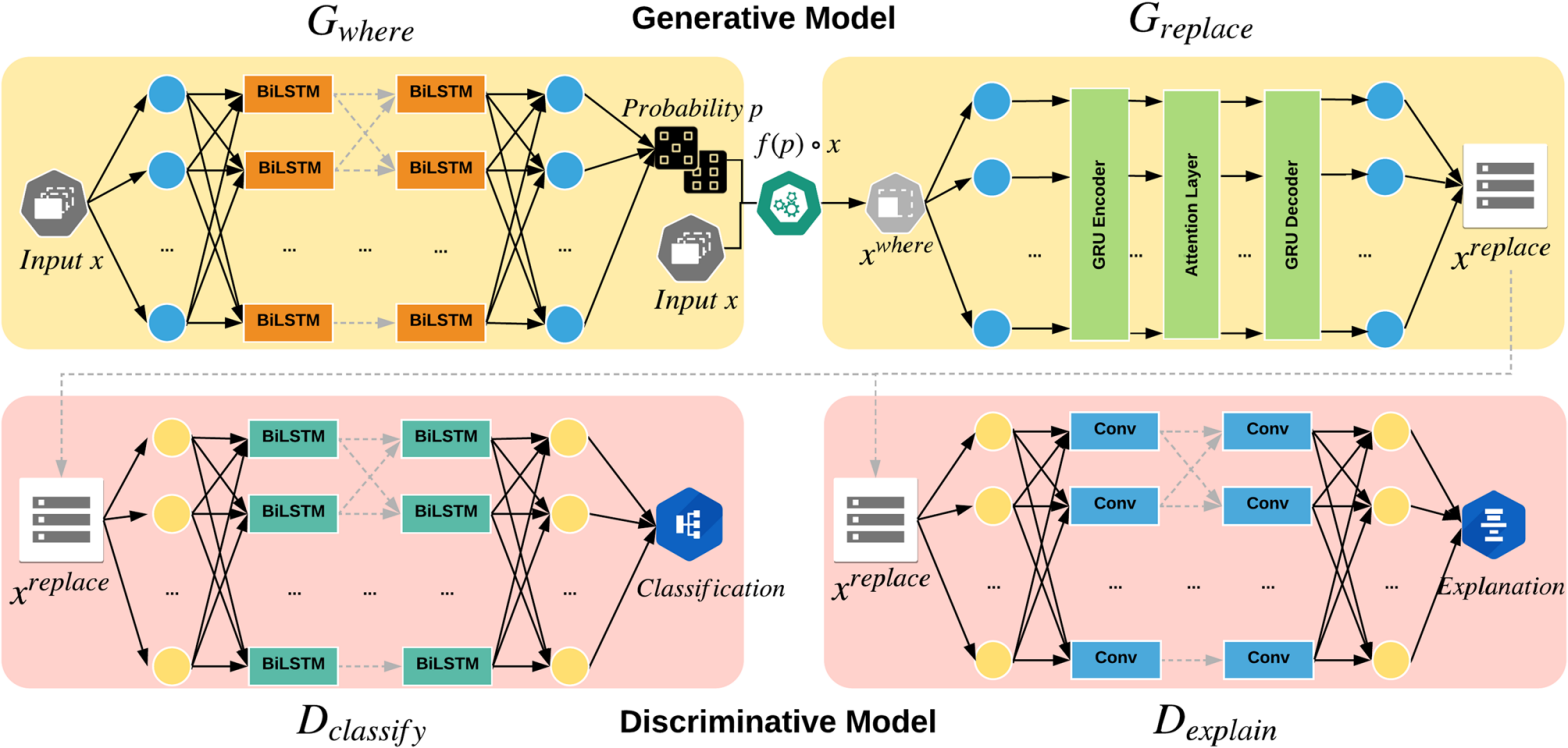
Our proposed framework. The generative model (shown on the left hand side) consists of two generators $G_{where}$ and $G_{replace}$. The discriminative model (shown on the right hand side) consists of two discriminators, namely $D_{explain}$ for explainability and $D_{classify}$ for classification.

### Our model—overview

Figure 2 shows the architecture of our proposed model. We have a layered generative model, which takes an input sequence and makes modifications intelligently; then a layered discriminative model to do classification and mutation detection. In rumor detection task, the generators must intelligently construct a rumor that appears like non-rumor to deceive the discriminators. Given a good lie usually has some truth in it, we choose to replace some of the tokens in the sequence and keep the majority to realize this goal. In our framework, two steps for intelligently replacing tokens in a sequence are: (1) determine where (i.e., which words / items in the sequence) to replace, and (2) choose what substitutes to use. $G_{where}$ and $G_{replace}$ are designed to realize these two steps. Having constructed the strong generators, the discriminators are designed to provide a defense mechanism. Through adversarial training, the generators and discriminators grow stronger together, in terms of generating and detecting rumors, respectively. In the rumor detection task, given a sentence, there are two questions that need to be answered: (1) is it a rumor or a non-rumor, and (2) if a rumor, which parts are problematic. $D_{classify}$ and $D_{explain}$ are designed to answer these two questions. We found that realizing two functions in one layer either in discriminative model or generative model hurts the performance. Hence, our framework was designed to embed a layered structure, and the detailed descriptions of the generative and discriminative model are as follows.

### Our model—generative model

The sequence generation task is done by the generative model: $G_{where}$ and $G_{replace}$. Given a human-generated real-world sequence input $\mathbf{x} = (x_1,x_2,\ldots ,x_M)$ with length *M*, such as a tweet-level sentence containing *M* words, $G_{where}$ outputs a probability vector $\mathbf{p} =(p_1,p_2,\ldots ,p_M)$ indicating the probabilities of each item $x_i$ ($i\in [1,M]$) to be replaced. $\mathbf{p}$ is applied to input $\mathbf{x}$ to construct a new sequence $\mathbf{x} ^{where}$ with some items replaced by blanks. For example, $x_2$ becomes a blank and then $\mathbf{x} ^{where} = (x_1,\_\ ,\ldots ,x_M)$.

$$\begin{aligned} \mathbf{x} ^{where} =f(\mathbf{p} )\circ \mathbf{x} = f(G_{where}(\mathbf{x} ))\circ \mathbf{x} , \end{aligned}$$

where $f(\cdot )$ binarizes the input based on a hyperparameter $N_{replace}$. It determines the percentage of the words to be replaced in a sentence. Operator $\circ$ works as follows. If $a = 1$, then $a\circ b = {\_}$. If $a = 0$, then $a\circ b = \_$. $G_{replace}$ is an encoder-decoder model with the attention mechanism. It takes $\mathbf{x} ^{where}$ and fills in the blank, then outputs a sequence $\mathbf{x} ^{replace} = (x_1,x^{replace}_2,\ldots ,x_M)$. The generative model is not fully differentiable because of the sampling operations on $G_{where}$ and $G_{replace}$. To train the generative model, we adopt policy gradients^40^ from RL to solve the non-differentiable issue.

### $G_{replace}$ GRU-based encoder

Gated Recurrent Units (GRUs)^41^ are the improved versions of standard RNNs that use update gates and reset gates to resolve the vanishing gradient problem of a standard RNN. In our GRU-based encoder, the hidden state $h_t$ is computed as $GRU_{encoder}(x^{where}_t,h_{t-1})$:

$$\begin{aligned} h_t= \,& {} (1-z_t)\odot h_{t-1}+z_t\odot h'_t,\\ z_t=\, & {} \sigma (W_{z}^{enc}x^{where}_t+U_{z}^{enc}h_{t-1}+b_{z}^{enc}),\\ h'_t=\, & {} tanh(W_{h}^{enc}x^{where}_t+U_{h}^{enc}(r_t\odot h_{t-1})+b_{h}^{enc}), \\ r_t= \,& {} \sigma (W_{r}^{enc}x^{where}_t+U_{r}^{enc}h_{t-1}+b_{r}^{enc}), \end{aligned}$$

where $W_{z}^{enc}$, $W_{h}^{enc}$, $b_{r}^{enc}$, $b_{z}^{enc}$, $b_{h}^{enc}$, $W_{r}^{enc}$, $U_{z}^{enc}$, $U_{h}^{enc}$ and $U_{r}^{enc}$ are encoder weight matrices. $\sigma (\cdot )$ is the sigmoid function. $\odot$ represents element-wise multiplication. *z*, *r*, and $h'$ are update gate, reset gate, and candidate activation in encoder, respectively.

### $G_{replace}$ GRU-based decoder with attention mechanism

Our encoder-decoder $G_{replace}$ utilizes attention mechanism^42^ to automatically search for parts of a sentence that are relevant to predicting the target word. The content vector $c_t$ summarizes all the information of words in a sentence. It depends on the annotations $h_t$ and is computed as a weighted sum of these $h_t$:

$$\begin{aligned} c_t = \sum _{j=1}^{M}\alpha _{tj}h_j,\quad \alpha _{tj} = \frac{exp(e_{tj})}{\sum _{k=1}^{M}exp(e_{tk})},\quad e_{tj} = a(s_{t-1},h_j), \end{aligned}$$

where $e_{tj}$ scores how well the inputs around position *j* and the output at position *t* match. Alignment model *a* is a neural network that jointly trained with all other components. The GRU decoder takes the previous target $y_{t-1}$ and the context vector $c_t$ as input, and utilizes GRU to compute the hidden state $s_t$ as $GRU_{decoder}(y_{t-1},s_{t-1},c_t)$:

$$\begin{aligned} s_t=\, & {} (1-z'_t)\odot s_{t-1}+z'_t\odot s'_t,\\ z'_t=\, & {} \sigma (W_{z}^{dec}y_{t-1}+U_{z}^{dec}s_{t-1}+C_{z}^{dec}c_t),\\ s'_t= \,& {} tanh(W_{s}^{dec}y_{t-1}+U_{s}^{dec}(r'_t\odot s_{t-1})+C_{s}^{dec}c_t),\\ r'_t= \,& {} \sigma (W_{r}^{dec}y_{t-1}+U_{r}^{dec}s_{t-1}+C_{r}^{dec}c_t), \end{aligned}$$

where $W_{z}^{dec}$, $W_{s}^{dec}$, $W_{r}^{dec}$, $U_{z}^{dec}$, $U_{s}^{dec}$, $U_{r}^{dec}$, $C_{z}^{dec}$, $C_{s}^{dec}$ and $C_{r}^{dec}$ are decoder weight matrices. $z'$, $r'$, and $s'$ are update gate, reset gate, and candidate activation in decoder, respectively. Through this attention-equipped encoder-decoder, $G_{replace}$ intelligently replaces items in sequences and outputs adversarial samples.

### Our model—discriminative model

The generated adversarial samples $\mathbf{x} ^{replace}$ combined with original data $\mathbf{x}$ are fed to the discriminative model. $D_{classify}$ and $D_{explain}$ are trained independently. We note that the two discriminators can depend on each other, but we have chosen to explore the dependency as part of our future work. $D_{classify}$ provides a probability in rumor detection, and $D_{explain}$ provides the probability of each word in the sentence being problematic. The explainability of our model is gained by adversarial training. We first insert adversarial items in the sequence, then train $D_{explain}$ to detect them. Through this technique, our model can not only classify data with existing patterns, but also classify sequences with unseen patterns that may appear in the future. Adversarial training improves the robustness and generalization ability of our model.

### Training

In the rumor detection task, a sequence $\mathbf{x}$ has a true label *Y* being either a rumor *R* or a non-rumor *N*. After manipulating the sequence $\mathbf{x}$, output of the generative model $\mathbf{x} ^{replace}$ is labeled as *R* since it is machine generated. The objective of a $\phi$-parameterized generative model is to mislead the $\theta$-parameterized discriminators. In our case, $D_{classify}^{\theta }(\mathbf{x} ^{replace})$ indicates how likely the generated $\mathbf{x} ^{replace}$ is classified as *N*. $D_{explain}^{\theta }(\mathbf{x} ^{replace})$ indicates how accurately $D^{\theta }_{explain}$ detects the replaced words in a sequence. The error attribution per time step is achieved naturally since $D_{explain}^{\theta }$ evaluates each token and therefore provides a fine-grained supervision signal to the generators. For example, a case where the generative model produces a sequence that deceives the discriminative model. Then the reward signal from $D_{explain}^{\theta }$ indicates how well the position of each replaced word contributes to the error result. The reward signal from $D_{classify}^{\theta }$ represents how well the combination of the position and the replaced word deceived the discriminator. The generative model is updated by applying a policy gradient on the received rewards from the discriminative model.

The rumor generation problem is defined as follows. Given a sequence $\mathbf{x}$, $G_{where}^{\phi }$ is used to produce a sequence of probabilities $\mathbf{p}$ indicating the replacing probability of each token in $\mathbf{x}$. $G_{replace}^{\phi }$ takes $\mathbf{x} ^{where}$ and produces a new sequence $\mathbf{x} ^{replace}$. This newly generated $\mathbf{x} ^{replace}$ is a sentence, part of which is replaced and labeled as *R*. At time step *t*, the state $\mathbf{s}$ consists of $\mathbf{s} ^{where}$ and $\mathbf{s} ^{replace}$. $\mathbf{s} ^{where} = (p_1,\ldots ,p_{t-1})$, $\mathbf{s} ^{replace} = (x^{replace}_1,\ldots ,x^{replace}_{t-1})$. The policy model $G_{where}^{\phi }(p_t|p_1,\ldots ,p_{t-1})$ and $G_{replace}^{\phi }(x^{replace}_t|x^{replace}_1,\ldots ,x^{replace}_{t-1})$ are stochastic. Following RL, $G^{\phi }_{where}$’s objective is to maximize its expected long-term reward:

$$\begin{aligned}&J_{where}(\phi ) = E[R_T|\mathbf{s} _0,\phi ]=\sum _{p_1} G^{\phi }_{where}(p_1|\mathbf{s} _0^{where})\cdot Q_{D^{\theta }}^{G^{\phi }}(\mathbf{s} _0^ {replace},\mathbf{a} ),\\&Q_{D^{\theta }}^{G^{\phi }}(\mathbf{s} _0^ {replace},\mathbf{a} ) = -D^{\theta }_{explain}(\mathbf{s} _0^{replace})+ D^{\theta }_{classify}(\mathbf{s} _0^{replace}), \end{aligned}$$

where $Q_{D^{\theta }}^{G^{\phi }}(\mathbf{s} _0,\mathbf{a} )$ is the accumulative reward following policy $G^{\phi }$ starting from state $\mathbf{s }_0 = \{\mathbf{s }_{0}^{where},\mathbf{s }^{replace}_{0}\}$. $-D^{\theta }_{explain}(\mathbf{s} ^ {replace})$ indicates how much the generative model misleads $D^{\theta }_{explain}$. $\mathbf{a}$ is an action set that contains output of both $G^{\phi }_{where}$ and $G^{\phi }_{replace}$. $R_T$ is the reward for a complete sequence. Similarly to $G^{\phi }_{where}$, $G^{\phi }_{replace}$ maximizes its expected long-term reward:

$$\begin{aligned} J_{replace}(\phi ) = \sum _{x^{replace}_1} G^{\phi }_{replace}(x^{replace}_1|\mathbf{s} _0^{replace})\cdot Q_{D^{\theta }}^{G^{\phi }}(\mathbf{s} _0^ {replace},\mathbf{a} ). \end{aligned}$$

We apply a discriminative model provided reward value to the generative model after the sequence is produced. The reason is that our $G^{\phi }_{replace}$ doesn’t need to generate each and every word in the sequence, but only fills a few blanks that are generated by $G^{\phi }_{where}$. Under this assumption, long-term reward is approximated by the reward gained after the whole sequence is finished.

The discriminative model and the generative model are updated alternately. The loss function of discriminative model is defined as follows:

$$\begin{aligned} L_{D}= & {} \lambda ^{explain}_{D}L^{explain}_{D}+ \lambda ^{classify}_{D}L^{classify}_{D},\\ L^{explain}_{D}= & {} -E_{y\sim f(G^{\phi }_{where}(\mathbf{x} ))} [ylog(D^{\theta }_{explain}(\mathbf{x} ^{replace}))+(1-y)log(1-D^{\theta }_{explain}(\mathbf{x} ^{replace}))]\\ L^{classify}_{D}= & {} -E_{y\sim Y} [ylog(D^{\theta }_{classify}(\mathbf{x} ^{replace}))+(1-y)log(1-D^{\theta }_{classify}(\mathbf{x} ^{replace}))] \end{aligned}$$

where $\lambda ^{explain}_{D}$ and $\lambda ^{classify}_{D}$ are the balancing parameters.

We adopt the training method in GANs to train the networks. In each epoch, the generative model and the discriminative model are updated alternately. Over-training the discriminators or the generators may result in a training failure. Thus hyper-parameters $\text {G}_{\text {STEP}}$ and $\text {D}_{\text {STEP}}$ are introduced to balance the training. In each epoch, the generators are trained $\text {G}_{\text {STEP}}$ times. Then discriminators are trained $\text {D}_{\text {STEP}}$ times.

### Experiment setup—model setup

Our model contains a layered generative model, $G_{where}$ and $G_{replace}$, and a layered discriminative model, $D_{explain}$ and $D_{classify}$. The architecture setup is as follows. $G_{where}$ consists of an RNN with two Bidirectional LSTM (BiLSTM) and one dense layer and seeks to determine the items in a sequence to be replaced. The $G_{where}$ architecture we used in all experiments has the architecture of EM-32-32-16-OUT, where EM, OUT represent embedding and output, respectively. $G_{replace}$ is an encoder-decoder with attention mechanism and is responsible for generating the substitutes for the items selected by $G_{where}$. The encoder has two GRU layers, and the decoder has two GRU layers equipped with attention mechanism. The architecture of $G_{replace}$ we used in all experiments is EM-64-64-EM-64-64-OUT. $D_{explain}$ has the same architecture as $G_{where}$ and is responsible for determine which items are problematic. $D_{classify}$ is a CNN with two convolutional layers followed by a dense layer. It is used for classification. The architecture we used in all experiments is EM-32-64-16-OUT.

### Experiment setup—data collection and augmentation

We evaluate our proposed model on a benchmark Twitter rumor detection dataset PHEME^43^, a misinformation/fake news dataset FMG^33^, and a splice site benchmark dataset NN269^44^. PHEME has two versions. PHEMEv5 contains 5792 tweets related to five news, 1972 of them are rumors and 3820 of them are non-rumors. PHEMEv9 contains 6411 tweets related to nine news, 2388 of them are rumors and 4023 of them are non-rumors. The maximum sequence length in PHEME is 40, and we pad the short sequences with zero padding. FMG dataset contains two parts corresponding to a veracity detection task (i.e., determine a news is true/false) and a provenance classification task (i.e., determine a news is real/fake), respectively. Input sequences with true label in veracity classification task are verified fact and false sequences are verified false statements. Input sequences with real label in provenance classification dataset are purely human-written sentences while the fake data are generated with pre-trained language models. We set the maximum sequence length as 1024 and 512 in true/false and real/fake tasks, respectively, and we pad the short sequences with zero padding and do post truncation on the text longer than length threshold. NN269 dataset contains 13231 splice site sequences. It has 6985 acceptor splice site sequences with length of 90 nucleotides, 5643 of them are positive *AP* and 1324 of them are negative *AN*. It also has 6246 donor splice site sequences with length of 15 nucleotides, 4922 of them are positive *DP* and 1324 of them are negative *DN*.

In rumor detection task, we generate a rumor/fake news/misinformation dataset denoted as PHEME’ (and FMG’), and then augment the original dataset with the generated sequences. Similarly, for the gene classification with mutation detection task, the proposed model generates a dataset NN269’ by replacing nine characters in acceptor sequences and three characters in donor sequences. We label the generated sequences by the following rules. In rumor detection with explanation task, (1) generated rumors based on PHEME are labeled as R (rumor) in 2-class classification (corresponds to results in Table 1); (2) in 4-class classification (corresponds to results in Table 5 and Table 6), if the input sequence $\mathbf{x}$ has label *Y*, then the output sequence $\mathbf{x} ^{replace}$ is labeled as $Y'$, indicating that $\mathbf{x} ^{replace}$ is from class *Y* but with modification. In gene mutation detection task, we follow the labeling rule described in (2), and the final classification output of our model is two-fold: *AP*, *AN* for acceptor, or *DP*, *DN* for donor. We merge the generated classes $AP'$, $AN'$ and $DP'$, $DN'$ with original classes to evaluate the noise resistance ability of our model. Given a sequence, our model can classify it into one of the known classes, although the sequence could either be clean or modified.

### Experiment setup—baseline description

In the rumor detection task, we compare our model with six popular rumor detectors: RNN with LSTM cells, CNN, VAE-LSTM, VAE-CNN, a contextual embedding model with data augmenting (DATA-AUG)^45^, and a GAN-based rumor detector (GAN-GRU)^13^. One of the strengths of our proposed model is that under the delicate layered structure that we designed, the choice of model structure affects the results but not significantly. To showcase this ability of the layered structure, we generate a variation of the proposed model by replacing $G_{replace}$ with a LSTM model as one baseline. It utilizes an LSTM-based encoder-decoder with architecture EM-32-32-EM-32-32-OUT as $G_{replace}$. Our model generates a set of sequences by substituting around $10\%$ of the items in original sequences. We pre-train the $D_{classify}$ by fixing the number of replacement $N_{replace} = 10\%$. We then freeze $D_{classify}$ and train the other three models. During training, we lower $N_{replace}$ from $50\%$ to $10\%$ to guarantee data balancing for $D_{explain}$ and better results in terms of explanations. All the embedding layers in the generators and discriminators are initialized with 50 dimension GloVe^46^ pre-trained vectors. Early stopping technique is applied during training. The generated data in the rumor task are labeled as *R*, and we denote this dataset as PHEME’. For fairness and consistency, we train baselines LSTM, CNN, VAE-LSTM, and VAE-CNN with PHEME and PHEME+PHEME’. For all baselines, we use two evaluation principles: (1) hold out $10\%$ of the data for model tuning, i.e., we split the dataset into training (with $90\%$ data) and test (with $10\%$ data) set. (2) Leave-one-out (L) principle, i.e., leave out one news for test, and train the models on other news. E.g., for PHEMEv5, where there are 5 events in the dataset, we pick 1 event as our test set and use the remaining 4 events as our training set. (Similarly, for PHEMEv9, where there are 9 events in the dataset, we pick 1 event as our test set and use the remaining 8 events as our training set.) Moreover, with L principle, we apply 5- and 9-fold cross validation for PHEMEv5 and PHEMEv9, respectively. Final results are calculated as the weighted average of all results. L principle constructs a realistic testing scenario and evaluates the rumor detection ability under new out-of-domain data. For DATA-AUG and GAN-GRU, we import the best results reported in their papers.

**Table 10:** Baselines’ architecture setup in both rumor detection task and gene classification with mutation detection task.

Model	Gene mutation detection task	Rumor detection task
LSTM	EM-LSTM(64)-LSTM(32)-DENSE(8)-OUT	EM-LSTM(32)-LSTM(16)-DENSE(8)-OUT
CNN	EM-CONV(32)-CONV(64)-DENSE(16)-OUT	EM-CONV(32)-CONV(16)-DENSE(8)-OUT
VAE-LSTM	LSTM(32)-LSTM(32)-DENSE(8)-OUT	LSTM(32)-LSTM(16)-DENSE(8)-OUT
VAE-CNN	CONV(32)-CONV(64)-DENSE(16)-OUT	CONV(32)-CONV(64)-DENSE(16)-OUT

In gene classification with mutation detection task we compare our models with five models: RNN with LSTM cells, CNN, VAE-LSTM, VAE-CNN, and the state-of-the-art splice site predictor EFFECT^47^. The first four baselines are trained under NN269+NN269’, and tested on both NN269+NN269’ and clean data NN269. We import EFFECT’s results from the original work^47^. The architectures of baselines LSTM, CNN, VAE-LSTM, and VAE-CNN used in both tasks are defined as in Table 10. VAE-LSTM and VAE-CNN use a pre-trained VAE followed by LSTM and CNN with the architectures we defined in Table 10. The VAE we pre-trained is a LSTM-based encoder-decoder. The encoder with architecture EM-32-32-32-OUT has two LSTM layers followed by a dense layer. The decoder has the architecture IN-32-32-OUT, where IN stands for input layer.
